# Crucial roles of the *CHRNB3–CHRNA6* gene cluster on chromosome 8 in nicotine dependence: update and subjects for future research

**DOI:** 10.1038/tp.2016.103

**Published:** 2016-06-21

**Authors:** L Wen, Z Yang, W Cui, M D Li

**Affiliations:** 1 State Key Laboratory for Diagnosis and Treatment of Infectious Diseases, Collaborative Innovation Center for Diagnosis and Treatment of Infectious Diseases, The First Affiliated Hospital, Zhejiang University School of Medicine, Hangzhou, China; 2 Air Center for Air Pollution and Health, Zhejiang University, Hangzhou, China; 3 Department of Psychiatry and Neurobehavioral Sciences, University of Virginia, Charlottesville, VA, USA

## Abstract

Cigarette smoking is a leading cause of preventable death throughout the world. Nicotine, the primary addictive compound in tobacco, plays a vital role in the initiation and maintenance of its use. Nicotine exerts its pharmacological roles through nicotinic acetylcholine receptors (nAChRs), which are ligand-gated ion channels consisting of five membrane-spanning subunits. Besides the *CHRNA4*, *CHRNB2* and *CHRNA5/A3/B4* cluster on chromosome 15, which has been investigated intensively, recent evidence from both genome-wide association studies and candidate gene-based association studies has revealed the crucial roles of the *CHRNB3*–*CHRNA6* gene cluster on chromosome 8 in nicotine dependence (ND). These studies demonstrate two distinct loci within this region. The first one is tagged by rs13277254, upstream of the *CHRNB3* gene, and the other is tagged by rs4952, a coding single nucleotide polymorphism in exon 5 of that gene. Functional studies by genetic manipulation in mice have shown that α6*-nAChRs, located in the ventral tegmental area (VTA), are of great importance in controlling nicotine self-administration. However, when the α6 subunit is selectively re-expressed in the VTA of the α6^−/−^ mouse by a lentiviral vector, the reinforcing property of nicotine is restored. To further determine the role of α6*-nAChRs in the process of nicotine-induced reward and withdrawal, genetic knock-in strains have been examined, which showed that replacement of Leu with Ser in the 9′ residue in the M2 domain of α6 produces nicotine-hypersensitive mice (α6 L9′S) with enhanced dopamine release. Moreover, nicotine-induced upregulation may be another ingredient in the pathology of nicotine addiction although the effect of chronic nicotine exposure on the expression of α6-containing receptors is controversial. To gain a better understanding of the pathological processes underlying ND and ND-related behaviors and to promote the development of effective smoking cessation therapies, we here present the most recent studies concerning the genetic effects of the *CHRNB3*–*CHRNA6* gene cluster in ND.

## Introduction

Cigarette smoking is one of the leading causes of preventable morbidity and death worldwide,^1^ being responsible for approximately five million deaths annually, mostly caused by smoking-related cancers and cardiovascular and respiratory diseases.^2^ Because of the continuous effort of legislation against tobacco smoking and public realization of its health consequences, the prevalence of cigarette smoking is lower than in the past but has been relatively stable since the mid-1990 s;^3^ however, the smoking cessation rate still is relatively low. In contrast to developed countries, the prevalence of smoking is still increasing in developing countries,^4^ further highlighting the urgency of developing new medicines for smoking cessation.

Tobacco use, including smoking initiation (SI) and nicotine dependence (ND), is a complex and multifactorial behavior determined by both genetic and environmental factors, as well as their interactions.^5^ Findings from twin studies have clearly demonstrated that genetics contributes to various smoking-related behaviors.^6, 7, 8^ After reviewing the reported genetic epidemiological studies on smoking behaviors, Sullivan and Kendler^9^ concluded that the heritability of SI and ND was 0.56 and 0.67, respectively, and Li *et al.*^10^ estimated the mean heritability of ND to be 0.59 in male smokers and 0.46 in female smokers.

There are >4000 ingredients in cigarette smoke,^11^ but the pharmacological effects of dependence are produced primarily by nicotine, which exerts its physiological roles through neuronal nicotinic acetylcholine receptors (nAChRs).^12^ The nAChRs, widely distributed in the central and peripheral nervous systems, are ligand-gated ion channels consisting of five membrane-spanning subunits^13^ that can modulate the release of neurotransmitters^14^ and mediate fast signal transmission at synapses.^15^ Binding of nicotine to nAChRs forms the molecular basis for the reward of nicotine and, eventually, the development of ND.

Different approaches have been employed, such as genetic, pharmacologic, and *in vitro* or *in vivo* functional studies, to link ND to one or more specific nAChR subunits.^16^ Because of the wide distribution of α4β2* (where ‘*' indicates additional subunits) nicotinic receptors in the brain and their high affinity for nicotine, a large body of research has focused primarily on these subunits.^17^ Recently, several genetic variants located in nAChR subunit encoding genes other than *CHRNA4* or *CHRNB2* were detected by genome-wide association studies (GWAS)^18^ and various candidate gene-based association and functional studies.^19, 20, 21^ For example, the most compelling SNP rs16969968 in *CHRNA5*,^22^ leading to an amino acid change in the position 398 (D398N) of the α5 subunit protein, has been consistently demonstrated to be a major biological contributor to ND.^20, 23^ For details on this part of the research, please refer to recent reviews.^22, 24, 25, 26^ It is thus believed that additional subunits or receptor subtypes are involved in the determination of different ND behaviors.

In this report, we review the evidence of genetic association between variants in the *CHRNB3–CHRNA6* gene cluster on chromosome 8 and ND or ND-related phenotypes. Further, we include the functional studies of α6 and β3 nAChR subunits in the etiologies of ND using genetically engineered knock out (KO) and knock-in (KI) mice.

## GWAS of the *CHRNB3–CHRNA6* gene cluster and ND

GWAS is an effective approach when one does not have prior knowledge of gene function(s) and pathological process of the disease of interest as a means to identify common susceptibility genetic variants for ND or other complex phenotypes of interest. Bierut *et al.*^27^ reported the first high-density association study on ND with the aim of identifying common genetic variants that contribute to the transition from occasional cigarette smoking to ND. The sample in this report consisted of 1050 heavy smokers, with a Fagerström Test for Nicotine Dependence (FTND) score of >4.0, and 879 light smokers, who showed no symptoms of ND. Among 2.4 million single nucleotide polymorphisms (SNPs) examined, multiple risk SNPs in the *CHRNB3–CHRNA6* cluster were identified, with the most compelling evidence for rs13277254 in *CHRNB3* (*P=*6.54 × 10^−5^). In addition, another SNP, rs6474413, in complete linkage disequilibrium with rs13277254 (*r*^2^=1) in the same gene, was identified, with a *P*-value of 9.36 × 10^−5^. These nominal associations (according to the current genome-wide significant threshold of 10^−8^) were subsequently replicated in a GWAS meta-analysis^21^ using the number of cigarettes smoked per day (CPD) to measure ND. Furthermore, Rice *et al.*^21, 28^ reported that *CHRNB3* was more strongly associated with FTND than with CPD, pointing out the importance of selecting an appropriate phenotype for association analysis. These authors carried out an independent GWAS with 1294 ND cases (defined by FTND score) and 2071 non-ND controls who had smoked at least 1 cigarette, revealing that the genetic locus most strongly associated with ND was rs1451240 in *CHRNB3* (odds ratio (OR) 0.65; *P=*2.4 × 10^−8^). This association was strengthened when combined in a subsequent meta-analysis with a previously published dataset^19^ (combined *P=*6.7 × 10^−16^; total *N=*4200). However, when CPD was used as an alternate phenotype, the association no longer reached genome-wide significance (*P=*0.0007). This result highlights that phenotype selection is important in genetic association study of ND. Although CPD is the most commonly used phenotype of smoking because of its easy measurement, available evidence supports the view that CPD is related to culture and ethnicity.^29^ Contrarily, the FTND score appeared to be a relatively invariant measure of ND.

## Candidate gene-based association studies of the *CHRNB3–CHRNA6* gene cluster with ND

Besides the latest application of GWAS, significant efforts have been made to identify susceptibility loci for ND and ND-related phenotypes through a candidate gene approach with both case–control and family-based designs. Because a set of common, highly correlated variants (*r*^2^=1) tagged by rs6474413 and rs13277254 in the *CHRNB3–CHRNA6* gene cluster has been associated with ND at genome-wide significance,^21, 27^ more attention has been paid to this region. So far, many candidate gene-based association studies have implicated various SNPs in the *CHRNB3–CHRNA6* cluster as having a significant effect on ND and ND-related phenotypes in multiple ethnic populations (Tables 1 and 2).

## Nicotine dependence

After analyzing 3713 SNPs in >300 candidate genes for their association with ND, Saccone *et al.*^19^ reported that rs6474413 (*P=*9.36 × 10^−5^) and rs10958726 (*P=*1.33 × 10^−4^) in *CHRNB3* are significantly associated with ND. Both SNPs are located in the putative 5′ promoter region of the gene, with rs6474413 being 2 kb away from the start codon and 15 kb from rs10958726. Because of the high linkage disequilibrium between the two SNPs, they may contribute to a single association signal. Using a sample of 1050 ND cases and 879 non-ND controls of European descent, the same population as used in the study of Saccone *et al.*,^19^ another study^30^ revealed a significant locus, tagged by rs13277254 at the 5′ end of *CHRNB3–CHRNA6*, that influences the transition from smoking to ND. This finding was replicated in the follow-up study,^38^ which considered peer smoking as a social environmental risk factor for smoking behavior.

**Figure 1:**
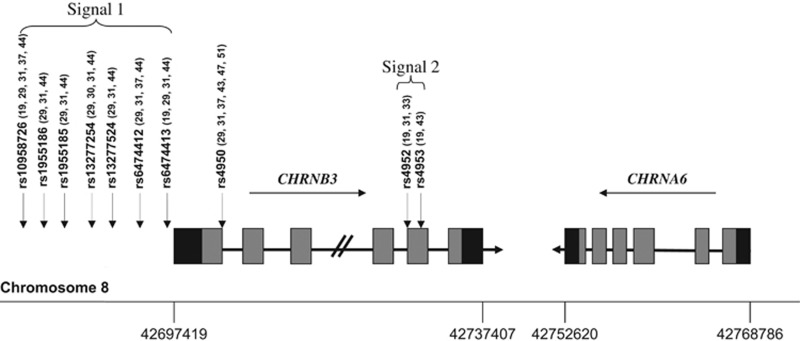
Schematic diagram of the human *CHRNB3–CHRNA6* gene cluster. Horizontal black arrows indicate the direction of transcription of each gene. Gray and black rectangles indicate exons and untranslated regions, respectively, while horizontal black lines represent introns (not drawn to scale). The genetic variants significantly associated with ND in EAs are shown by vertical arrows, which represent two distinct signals. EA, European-American; ND, nicotine dependence.

On the basis of the previous association results of a high-density study covering the complete family of 16 *CHRN* genes in a population of European ancestry, Saccone *et al.*^30^ extended their research to determine whether variants in the *CHRNB3–CHRNA6* gene cluster also are associated with ND in African-Americans (AAs).^33^ They did not detect any associated SNP in their AA sample with a sample size of 710. It was suggested that there might exist at least two distinct loci in the *CHRNB3–CHRNA6* gene cluster that are associated with ND in European Americans (EAs). The first one was tagged by rs13277254, upstream of the gene cluster, together with additional associated SNPs in this region constituted Signal 1. Signal 2 was tagged by rs4952, the only known coding SNP in the exon 5 of *CHRNB3*, which had a low correlation with rs13277254 (Figure 1).

There also exist many other common variants in the *CHRNB3–CHRNA6* gene cluster that show a significant association with ND in multiple ethnic populations, including Han Chinese,^40^ AAs,^39^ EAs,^43, 44^ and Israelis.^46^ We performed a meta-analysis of variants in *CHRNB3* in relation to ND by combining data from the studies of subjects of different ethnicities.^34^ Although allele frequencies in AAs were different from those in EAs and subjects of Asian ancestry, where the last two ethnic samples appeared to be similar, we found that the genetic effect of seven SNPs in *CHRNB3* are in the same direction among the three populations. More importantly, all these SNPs showed a significant association with ND. However, because of the different genetic structures of various ancestries, inconsistent results were found at the SNP level. We detected only four of seven SNPs in the samples of African origin, whereas the associations of all SNPs in the samples of European and Asian ancestry were significant.^34^ In contrast, none of these SNPs was reported to be associated with ND among three other studies in Finnish,^47^ Swiss^48^ and Czech^49^ populations. Hubacek^49^ attributed this discrepancy partly to socioeconomic status in that the prevalence of smoking was higher in post-Communist countries than in western European countries, and this fact could mask the real effect of each SNP. Thus, further replication studies in additional independent samples of different origins are warranted.

## ND-related phenotypes

The early-subjective response to tobacco smoking is a subphenotype of SI, which can predict later persistence of smoking and addiction. DiFranza *et al.*^50^ reported that greater sensitivity to nicotine during early-smoking attempts, as manifested by relaxation, dizziness or nausea, was a determinant of later ND. Pomerleau *et al.*^51^ found that smokers who felt a pleasurable buzz during early smoking smoked much later than those who did not. Thus, it was reasonable to assume that genes, especially *CHRN*, associated with ND might also play a role in early-subjective responses to tobacco.

The first report concerning the association between the variants in *CHRNB3–CHRNA6* and subjective responses to tobacco was published by Zeiger *et al.*^31^ using as subjects 1056 ethnically diverse adolescents and a separate community sample of 1524 families. The most significant associations were found between two *CHRNB3* SNPs (that is, rs4950 and rs13280604) and three subjective response factors to initial tobacco use (adverse, negative physical and positive). Since then, several studies^36, 42, 52^ have examined the association between variants in the *CHRNB3–CHRNA6* gene cluster and dizziness at first inhalation of cigarette smoke. Although both Ehringer *et al.*^36^ and Pedneault *et al.*^42^ have detected associations with several SNPs in the putative promoter region of *CHRNB3* and *CHRNA6*, Hoft *et al.*^52^ did not, which might be attributable to the small sample and the discrepancy of the phenotypic assessment tools used in these studies.

Apart from early-subjective responses to tobacco, there exist many other ND-related phenotypes where the *CHRNB3–CHRNA6* gene cluster may play an important role, such as smoking status (never smoking versus ever smoking),^35^ smoking trajectories from early adolescence to adulthood,^45^ and various ND endophenotypes such as ‘novelty seeking'^53^ or ‘drive.'^37^ In addition, smoking cessation is of great interest, because it is the ultimate goal of studying tobacco addiction and any other smoking-related phenotypes. Hoft *et al.*^32^ examined the association of SNPs in the CHRNB3–CHRNA6 cluster with quit attempts in a nationally representative sample of households, which revealed that three SNPs upstream of *CHRNB3* (that is, rs7004381, rs4950, rs13280604) and an SNP (rs2304297) in the 3′-region of *CHRNA6* were significantly associated with the number of unsuccessful quit attempts in Caucasians. Further, Fletcher *et al.*^41^ provided novel evidence of the importance of genetics in explaining different responses to tobacco taxation policy. These investigators found that individuals with the protective G/G polymorphism of rs2304297 in *CHRNA6* responded to high tobacco taxation, which may help with abstention, whereas others had no response. The inability of this tobacco control policy (high taxation) to reduce the use of cigarettes in individuals with the C/C genotype suggests that alternative methods might be needed to increase smoking cessation in this population.

## Analysis of rare variants in the *CHRNB3–CHRNA6* gene cluster

Both GWAS and candidate gene-based association studies have identified multiple common variants in the *CHRNB3–CHRNA6* gene cluster that contribute to ND and ND-related phenotypes. However, the role of rare variants of this cluster in ND has rarely been studied, largely because the extremely low minor allele frequency (MAF) poses great difficulties in ensuring adequate statistical power. The only study of this topic was carried out by Haller *et al.*,^54^ in which a DNA-pooling approach was used to sequence the coding and flanking regions of *CHRNA6* and *CHRNB3* in AA and EA ND smokers or smokers without any ND symptom (for the AAs, two case pools and two control pools; for the EAs, one case pool and one control pool). In contrast to another study performed by the same group,^55^ which showed that rare missense variants in *CHRNB3* were associated with a risk of alcohol and cocaine dependence, there was no evidence supporting the role of the same variants in ND.^54, 55^

Despite the absence of genetic association data for most SNPs, functional studies conducted by us^56^ indicated that rare variants in the hα6 subunit gene play a vital role in the etiology of ND. Although missense variations such as Asp57Asn (rs149966755) and Ser156Arg (rs373147726), Asn171Lys (rs79945499) compromises the function of hα6*-nAChRs heterologously expressed in *Xenopus* oocytes, the nicotine sensitivity of these receptors is marginally or significantly increased by introducing Arg96His (rs188620180), Ala184Asp (rs200745568), Asp199Tyr (rs372469952) or Ser233Cys (rs369966241) variations into the hα6 subunit gene. Greater sensitivity to activation by agonists (nicotine or ACh) may result in a lower risk of ND, whereas reduced sensitivity increases the risk.^57^ Individuals displaying altered α6*-nAChR pharmacology as a result of rare variants in *CHRNA6* are expected to exhibit different responses to cigarette smoking.

Because rare variants (defined as those having an MAF of <1%), together with copy-number variants and small insertion/deletion polymorphisms (indels) constitute the majority of human genetic variations, they might contribute, at least partly, to the missing heritability of ND. Thus, we need to take rare variants into consideration when studying ND-related phenotypes, especially rare missense functional variants.

## Functional studies of the β3 and α6 subunits by genetic manipulation in rodents

As described above, numerous genetic studies have revealed a highly significant association between variants in the *CHRNB3–CHRNA6* gene cluster and increased vulnerability to ND,^21, 27, 28^ which generates a need to explore the underlying mechanisms. However, to date, few pharmacologic ligands have been developed that can selectively target specific nAChR subtypes. Therefore, to understand the contribution of α6 and β3 subunits to ND susceptibility *in vivo*, and to circumvent the problem mentioned above, together with the difficulty associated with α6*-nAChRs *in vitro* expression genetic manipulation in mice becomes highly valuable. These manipulations generally include preventing the expression of the α6 or β3-subunit (KO) and replacing it with hyperactive derivatives (KI).

More attention has been paid to α6*- and β3*-nAChRs since the demonstration that these subunits exhibit an expression pattern restricted mainly to catecholaminergic and visual system neurons.^58, 59, 60, 61^ By using transgenic mice expressing the α6 subunit fused with green fluorescent protein, the α6 subunit was found to be highly and selectively expressed in the ventral tegmental area (VTA) and substantia nigra pars compacta, important regions for reinforcement of nicotine use,^62, 63^ with functional expression also in the locus coeruleus and retinal ganglion cells.^64, 65^ Immunoprecipitation and high-affinity [^125^I]α-conotoxinMII (αCtxMII)-binding studies showed that α6β2β3* and α6α4β2β3* pentamers are the predominant α6*-nAChRs in the striatum.^66, 67^ Furthermore, the gene encoding the β3-subunit, which is adjacent to *CHRNA6* (Figure 1), usually is co-expressed with α6. Because of the accessory role of the β3-subunit, it cannot form an acetylcholine-binding site, although it has an essential role in α6*-nAChR biogenesis and function.^68, 69^ Gotti *et al.*^69^ discovered that β3-subunit deletion dramatically reduced, but did not eliminate, α6*-nAChRs expression in the DA cell body (VTA) and terminal region (striatum), suggesting the importance of β3 for the correct assembly, stability and transport of α6-containing receptors in dopaminergic neurons. In addition, a study conducted by Cui *et al.*^68^ demonstrated that disruption of the β3 gene does not affect expression of mRNA for α6 and other subunits in the same brain areas. They also found that β3-KO mice have altered locomotor activity and prepulse inhibition of acoustic startle responses, behaviors that are regulated in part by nigrostriatal and mesolimbic dopaminergic neurotransmission. Knowledge of these alterations is supported by the evidence that a population of β3-dependent nAChRs, which are sensitive to inhibition by αCTxMII, modulate striatal dopamine release.^68^ In addition, Kamens *et al.*^70^ showed that the protective variant rs6474413 from human studies reduced expression of the *CHRNB3* subunit, and decreased β3 gene expression resulted in reduced nicotine intake in mice.

The α6-null mice grow normally and show no obvious developmental, neurologic or behavior deficits.^66, 71^ By using autoradiography, Champtiaux *et al.*^71^ found complete disappearance of [^125^I] α-CtxMII binding in both midbrain dopamergic neurons and the visual system after deleting the α6 subunit, indicating that α6 is an essential component of the native-binding site of this toxin. Another study^72^ has shown the central role of α6 in the VTA in acute nicotine reinforcement.

**Figure 2:**
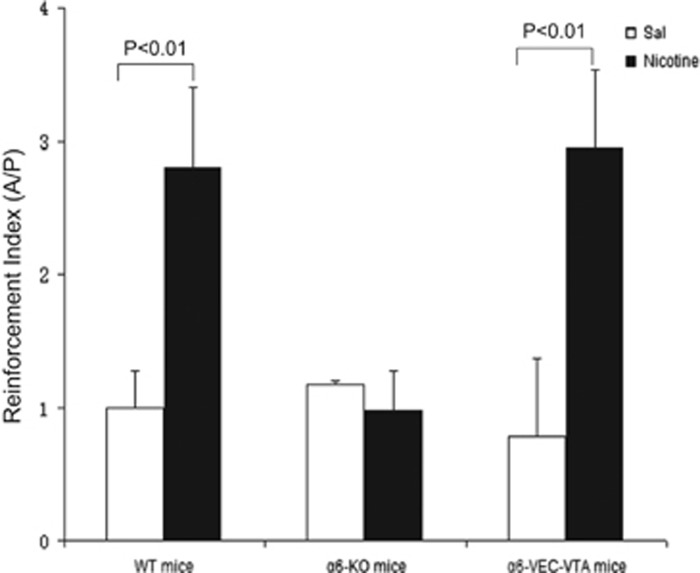
Nicotine intravenous self-administration in WT and α6-VEC-VTA mice, but not in α6-KO mice. Data are presented as mean (±s.e.m.) reinforcement index (that is, ratio of the cumulative nose pokes (NPS) of the active mice with respect to yoked control passive mice over the 30- min session in each group). The dose of nicotine was 26.3 μg kg^−1^ per inf. *P<*0.01 indicates statistically significant differences between nicotine-treated and saline control groups. The data used in the figure are adapted from the study by Pons *et al.*^72^ KO, knock out; VTA, ventral tegmental area; WT, wild type*.*

**Figure 3:**
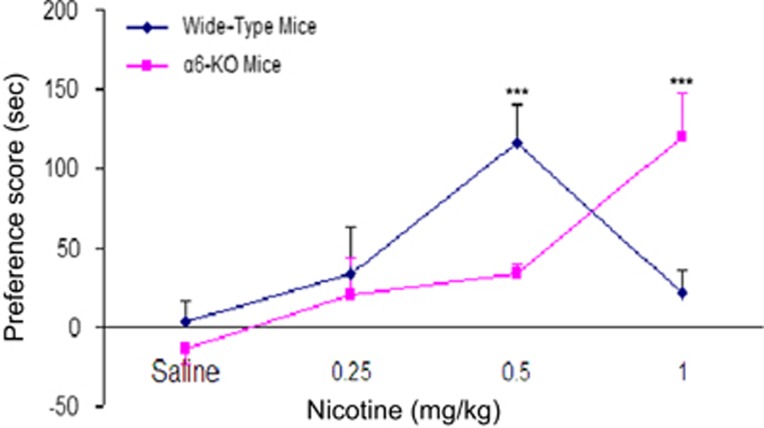
Crucial role of α6* nAChRs in the rewarding effects of nicotine based on conditioned place preference (CPP). The α6-KO mice exhibited a rightward shift in the nicotine dose–response curve compared with WT littermates. Data are presented as mean (±s.e.m.) preference score (sec). ***P<0.001. The data are adapted from the study by Sanjakdar *et al.*^77^ KO, knock out; nAChR, nicotinic acetylcholine receptor; WT, wild type.

There are two primary strategies for measuring the reinforcing effect of nicotine: one is intravenous or intracranial nicotine self-administration (SA)^73, 74^ and the other is nicotine-induced conditioned place preference (CPP).^75^ The SA paradigm is usually conducted in 30 min with matched animal pairs placed in the experimental boxes, with one animal defined as active and the other as passive. Each nose-poke (NP) by the active mouse activates the computer-operated syringe pump delivering either nicotine or saline to both the active and passive animals, while NPs of the passive mouse are recoded with no scheduled consequences. By calculating the ratio between the number of responses (NPs) of the active and passive mouse, the reinforcing effects of nicotine can be determined. When tested in this way, α6-WT mice self-administered nicotine in a unit dose of 26.3 μg kg^−1^ per infusion (inf), whereas their α6-KO drug-naive littermates did not. The α6-KO animals did not self-administer nicotine even in an extensive range of lower (8.7-17.5 μg kg^−1^ per inf) and higher (35-52.6 μg kg^−1^ per inf) doses. Importantly, when the α6 subunit was selectively re-expressed in the VTA of α6^−/−^ mice using a lentiviral vector, the reinforcement property of nicotine was restored (Figure 2).^72^ In intracranial SA experiments where learning is required, α6-KO mice showed a trend (although it was not significant) toward reduced nicotine SA compared with wild-type (WT) control mice.^76^ These findings demonstrate that the α6 subunit in the VTA is necessary for maintaining nicotine SA. By employing the latter model, Sanjakdar *et al.*^77^ showed that nicotine displayed a typical inverted U-shaped CPP response curve in the WT mice. Although the dose of 0.5 mg kg^−1^ nicotine led to a significant CPP in the WT mice, it failed to produce a CPP response in α6-KO mice. In contrast, the higher nicotine dose of 1.0 mg kg^−1^ resulted in preference scores in α6-KO mice, which were significantly higher than in α6-WT littermates (Figure 3). The α6-KO mice exhibit a rightward shift in the nicotine dose–response curve compared with WT mice, indicating that the rewarding effect of nicotine is mediated by α6*-nAChRs. Pharmacologic blockade of the α6 subunit by selective antagonists (for example, α-contoxinMII) attenuates nicotine-induced CPP,^77, 78^ further supporting the vital role of α6 in the nicotine reinforcement.

Although the KO mice model is an essential research tool for understanding the mechanisms of ND, it typically allows addressing only questions of necessity, not sufficiency. To fully understand the diverse roles of different subunits or subtypes in the process of nicotine-induced reward and withdrawal, genetic KI strains have been developed. Replacement of ‘Leu' with ‘Ser' in the 9′ residue in the M2 domain of the α6 subunit produces nicotine-hypersensitive mice. These α6 L9′S strains show hyperactive locomotion and fail to habituate to a home cage, a novel environment or reduced wheel rotations,^79, 80, 81, 82^ which is consistent with enhanced dopamine neuron firing and release.^79, 80, 82, 83^ In addition, by crossing the α4-KO mice with α6L9′S strains, it was found that the hyperactive effects caused by the gain-of-function mutation are mediated by α6α4* pentamers, because α6L9′S mice lacking the α4 subunit display essentially normal behavior.^80^ Together, these studies demonstrate that α6L9′S mice are valuable in investigating the role of the α6 subunit in ND-related behaviors.

## Effect of chronic nicotine exposure on the expression of α6-containing nAChRs

Nicotine, like other substances of abuse, enhances dopamine transmission in the mesolimbic dopamine pathway, which is thought to play a critical role in the reinforcing effects that maintain smoking behaviors. Many studies on the rewarding effects of nicotine employed an acute administration approach. However, because smoking is a chronic behavior leading to long-term adaptive changes in the brain, knowledge of these chronic changes is essential for understanding ND and implementing measures that cause smoking cessation. Therefore, if genetic manipulation of nAChR genes in mouse KO or KI models represents a powerful research tool for identification of the particular contribution of specific receptor subunits to ND susceptibility, chronic nicotine treatment *in vivo* or *in vitro*, which mimics the process of smoking in humans, is a valuable strategy.

After long-term nicotine exposure, high-affinity agonist binding to nAChRs in the central nervous system increases in both animal^84, 85^ and human^86^ brains. This process, termed ‘nicotine-induced upregulation,' ^87^ may be involved in the pathology of nicotine addiction. An increase in [^3^H]Ach-binding sites was reported in the brains of smokers compared with non-smokers.^88^ The essence of nAChRs upregulation is more related to greater receptor numbers than to augmentation of receptor affinity for nicotine.^89^ Furthermore, a hypothesis that nicotine acts as a pharmacologic chaperone to enhance a critical step inside the cell during the maturation of nAChRs has gained support recently.^90^ Specifically, nicotine binding to partially assembled nAChRs induces conformations that assemble more efficiently. This could be a compensatory response following desensitization of neuronal AChRs after chronic nicotine exposure.^91, 92^

Accumulating studies have consistently observed upregulation by radiolabeled epibatidine, which identified several nAChR subtypes in numerous brain regions after various nicotine treatments, including injection by osmotic minipumps or jugular cannula and infusion in drinking water.^85, 93, 94, 95, 96^ Using [^125^I]epibatidine, A-85380, and cytosine, Nguten *et al.*^97^ demonstrated that chronic exposure to nicotine upregulates α4β2-containing receptors while having little effect on other nAChR subtypes. Nevertheless, α4β2*-nAChRs, with a wide distribution in the brain and high affinity for nicotine, clearly become desensitized at an early stage of smoking behavior and thus do not function for most of the day in smokers. Despite the clarity of α4β2*-nAChR upregulation, it is not sufficient to explain continued smoking throughout the day.^16, 98^ On the other hand, nAChRs with low affinity for nicotine (for example, α7, α6) are not susceptible to rapid saturation and might play an important role in continued smoking. Besides α4β2-containing receptors, other diverse populations of nAChRs, such as α6β2* and α7*, have been identified in the mesolimbic dopamine pathway. These findings shed light on the vital importance of research on the upregulation of other nAChRs.

Unlike the situation with α4β2*-nAChRs, upregulation of α6-containing receptors in response to chronic nicotine exposure is controversial.^99^ There have been reports of upregulation, downregulation and no change in *in vitro* and *in vivo* experiments (Table 3). Upregulation of α6β2*- or α6β2β3*-nAChRs by incubation with nicotine was observed in cultured cell lines,^100, 101, 102^ although functional expression of α6-containing receptors in a heterologous expression system proved to be difficult until some specific strategies were used, such as chimeras, gain-of-function mutagenesis and so on. Unfortunately, in rodents, although Nguyen *et al.*^97^ and Parker *et al.*^103^ suggested upregulation of α6*-nAChRs in the nucleus accumbens, several other research groups^104, 105, 106, 107, 108^ observed downregulation in the striatum. Interestingly, Perez *et al.*^107^ showed, by using the novel α-CtxMII analog E11A in α4-KO mice, that nicotine administration in the drinking water for 2 weeks increased the α6 (non-α4) β2*-nAChR population in the striatum, contrary to the reduction of total α6β2* subtypes in WT littermates. This leads us to hypothesize that α6α4β2* contributes to the downregulation in the striatum. Furthermore, in non-human primates such as the squirrel monkey, nicotine in the drinking water with a final concentration of 650 μg ml^−1^ for >6 months did not significantly change the α6β2*-nAChR-binding site^109, 110, 111^ except in the study conducted by McCallum *et al.*^112^ This effect might be caused by region-specific actions, because earlier studies concentrated mainly on the nucleus accumbens, whereas the later ones focused on the striatum. Analyses in other reward-related regions of the brain also were performed, but this work has yielded no clear results or conclusions.^102, 103, 113^

Several factors may account for these disparate findings. First, different nicotine treatment regimens with different concentrations of nicotine and exposure time were used. The importance of such changes is supported by evidence that α6β2β3*-nAChR showed upregulation after 50 nm nicotine treatment but downregulation with 500 nm nicotine.^102^ Second, different species/cell lines, brain regions and α6-containing subtypes may play a role in the inconsistent results. Last but not least, heterogeneity of the detection methods is an influencing factor, implying the urgency of developing more subunit-specific agonists and antibodies.

## Conclusions and future research

In this report, we have summarized several lines of evidence that support the involvement of the *CHRNB3–CHRNA6* gene cluster in ND. A multitude of genetic studies (GWAS and candidate gene-based association studies) analyzing various ND phenotypes have implicated variants in this gene cluster in the development of ND. The most compelling evidence is for SNPs rs13277254 and rs6474413 in *CHRNB3*, as well as rs10958726 and rs1955186 within this same signal. However, not much has been found specifically for the *CHRNA6* subunit gene, contrary to its vital role in maintaining ND as demonstrated with functional studies. These findings reveal only a small fraction of variants, that is, these known polymorphisms have small effects and can explain only a small proportion of the heritability of smoking-related behaviors. Therefore, additional loci (especially rare variants) need to be identified. Furthermore, despite the inconsistent results, it is important to study the genetics of ND in diverse populations. Differences in genetic architectures and allele frequencies in different ethnic populations can help assign statistically significant signals to potentially causal variants.

Genetic modification of the *CHRNB3* and *CHRNA6* in mice is a valuable approach to evaluate the contribution of each subunit to ND susceptibility. The use of KO mice has displayed various behavioral phenotypes related to ND. For example, α6-KO mice do not self-administer nicotine, unlike their WT counterparts. In addition, studies in α6-hypersensitive mice (KI mice) are powerful in identifying compounds that activate or antagonize α6*-nAChRs as a means to improve the development of drugs for smoking cessation. Nevertheless, this approach is limited in the *in vivo* or *in vitro* studies focusing on elucidating the functional consequences of different SNPs. This level of investigation will provide significant insights into how genetic variations in humans underlie individual differences in the reinforcement, aversion and withdrawal of nicotine. There exist significant differences in the pharmacologic properties of the α6 and β3 subunits, such as receptor upregulation after chronic nicotine treatment and differences among subtypes and brain regions. It remains to be determined how nicotine regulates the expression of α6*-nAChRs. Inconsistent results from different studies were likely a consequence of the unpredictable behavior in heterologous expression systems. Functional expression of WT α6*-nAChRs is difficult to achieve unless some modifications have been adopted, for instance, subunit chimeras, concatameric subunits and point mutagenesis of the α6 or β3 subunits. In spite of the significant progress, there still are many obstacles to be overcome. That may be why conflicting results concerning upregulation of α6-containing receptors occur in relatively few studies. Thus, advancing the heterologous expression of α6* receptors should be another focus of future research.

## Figures and Tables

**Table 1:** Replicated SNPs in the *CHRNB3* gene cluster associated with ND-related behaviors

*dbSNP ID*	*Sample origin*	*Sample size*	*Phenotype*	*Odds ratio or* β*-value*	*Reported* P*-value*	*Reference*
rs4950	EA and Australian	1929	ND (FTND)	1.38	0.0001	30
	Ethnically diverse	1056	Subjective responses to tobacco (adverse, negative physical, positive)	4.88 8.13 12.25	0.02, 0.004, <0.001	31
	Ethnically diverse	1524 families	Subjective responses to tobacco	NA	0.043	31
	Caucasian, AA and Hispanic	1051	Quit attempts	NA	0.021	32
	Caucasian, AA and Hispanic	295	ND	4.62	0.007	32
	EA	2062	ND	0.78	0.00143	33
	EA, AA and Asian (meta-analysis)	22 654	ND	0.1343	1.08E−05	34
	Ashkenazi	591	Smoking status	1.94	9.8E−05	35
						
rs10958726	EA and Australian	1929	ND (FTND)	NA	1.33E−04	19
	EA and Australian	1929	ND (FTND)	1.38	9.636E−05	30
	EA	1600	Early subjective response to tobacco (dizziness)	−0.126	0.005	36
	EA	2062	ND	0.77	0.00113	33
	EA, AA and Asian (meta-analysis)	22 654	ND	0.1546	1.24E−07	34
						
rs13280604	Ethnically diverse	1056	Subjective responses to tobacco (adverse, negative physical, positive)	5.00 12.61	0.03, 0.001, <0.001	31
	Ethnically diverse	1524 families	Subjective responses to tobacco	NA	0.011	31
	Caucasian, AA and Hispanic	1051	Quit attempts	NA	0.024	32
	Caucasian, AA and Hispanic	295	ND	4.67	0.006	32
	EA, AA and Asian (meta-analysis)	22 654	ND	0.1362	7.77E−06	34
	Korean	576	NDSS (drive)	NA	0.03	37
						
rs6474413	EA and Australian	1929	ND (FTND)	NA	9.36E−05	19
	EA and Australian	1929	ND (FTND)	1.39	6.260E−05	30
	EA	1600	Early subjective response to tobacco (dizziness)	−0.114	0.011	36
	EA	2062	ND	0.77	9.26E−04	33
						
rs13277254	EA and Australian	1929	ND (FTND)	1.4	4.022E−05	30
	EA	1600	Early subjective response to tobacco (dizziness)	−0.122	0.007	36
	EA	2038	ND (FTND)	0.79	0.004	38
	EA	2062	ND	0.76	6.25E−04	33
						
rs6474412	EA and Australian	1929	ND (FTND)	1.38	1.126E−04	30
	EA	1600	Early subjective response to tobacco (dizziness)	−0.111	0.014	36
	EA	2062	ND	0.78	0.00137	33
	EA, AA and Asian (meta-analysis)	22 654	ND	0.1548	5.34E−07	34
						
rs4952	EA and Australian	1929	ND (FTND)	NA	0.0163	19
	EA and AA	2772	ND	NA	0.00881	33
	EA and AA (meta-analysis)	5092	ND (FTND)	0.72	0.02	39
						
rs1955186	EA and Australian	1929	ND (FTND)	1.38	8.252E−05	30
	EA	1600	Early subjective response to tobacco (dizziness)	−0.119	0.009	36
	EA	2062	ND	0.77	7.38E−04	33
						
rs1955185	EA and Australian	1929	ND (FTND)	1.38	1.010E−04	30
	EA	1600	Early subjective response to tobacco (dizziness)	−0.118	0.009	36
	EA	2062	ND	0.78	0.00117	33
						
rs13277524	EA and Australian	1929	ND (FTND)	1.39	6.043E−05	30
	EA	1600	Early subjective response to tobacco (dizziness)	−0.121	0.007	36
	EA	2062	ND	0.77	7.78E−04	33
						
rs4953	EA and Australian	1929	ND (FTND)	NA	0.0162	19
	Ethnically diverse	1056	Subjective responses to tobacco (adverse)	4.16	0.04	31
						
rs4954	Han Chinese	48	ND (FTND)	2.18	4.25E−07	40
	Korean	576	NDSS (drive)	NA	0.02	37

Abbreviations: AA, African-American; EA, European-American; FTND, Fagerström Test for Nicotine Dependence; NA, not available; ND, nicotine dependence; NDSS, nicotine-dependence syndrome scale; SNP, single nucleotide polymorphism.

**Table 2:** Replicated SNPs in the *CHRNA6* gene cluster associated with ND-related behaviors

*dbSNP ID*	*Sample origin*	*Sample size*	*Phenotype*	*Odds ratio or* β*-value*	*Reported* P*-value*	*Reference*
rs2304297	EA and Australian	1929	ND (FTND)	NA	0.00691	19
	Ethnically diverse	1056	Subjective responses to tobacco (positive)	0.170	0.003	31
	Caucasian, AA and Hispanic	1051	Quit attempts	NA	0.0044	32
	Mixed ethnic samples	6178	Response to tobacco taxation policy	−0.032	0.018	41
	Canadian	356	Dizziness at first inhalation of cigarette smoke	0.59	0.0057	42
						
rs7828365	American	2847	ND (CPD)	0.84	0.036	43
	Canadian	356	Dizziness at first inhalation of cigarette smoke	0.58	0.0293	42
						
rs9298628	Korean	576	NDSS (drive)	NA	0.02	37
	EA	2428	ND (FTND)	NA	2.18E−04	44
	EA and AA (meta-analysis)	7186	ND (FTND)	NA	0.00498	44
						
rs892413	Ethnically diverse	935	Smoking trajectories	−1.12	<0.001	45
	EA	1730	ND (CPD)	NA	0.00769	44
	EA	2428	ND (FTND)	NA	5.30E−04	44
	EA and AA (meta-analysis)	7186	ND (FTND)	NA	0.00311	44

Abbreviations: AA, African-American; CPD, cigarettes smoked per day; EA, European-American; FTND, Fagerström Test for Nicotine Dependence; NA, not available; ND, nicotine dependence; NDSS, nicotine-dependence syndrome scale; SNP, single nucleotide polymorphism.

**Table 3:** Effect on the expression of α6- and β3-containing nAChRs by chronic nicotine exposure

*Change*	*Species/cells*	*Treatment/dose*	*Brain region*	*Subtype*	*Reference*
Upregulation	Rat	Injection; 6.0 mg kg^−1^ per day; 2 weeks	NAcc; SC	α6β2*	97
		Injection; 1.5 mg kg^−1^ per day; 18 day	NAcc; VTA/SN; CPu; Thal	α6*	103
					
	Mouse	Injection; 0.4 mg kg^−1^ per h; 10 day	VTA/SNc	α6*	102
		Injection; 2 mg kg^−1^ per h; 10 day	VTA/SNc; mHb; SC	α6*	102
		Oral; 300 μg ml^−1^; 2 weeks	Str	α6(nonα4)β2*	107
					
	HEK tsA201 cell	Incubation; 100 μm; overnight	—	α6β2* α6β2β3*α6β4; α6β4β3*	100
		Incubation; 30 μm; 24 h	—	α6β2*	101
					
	Neuro-2a cell	Incubation; 50 μm; 24 h	—	α6β2β3*	102
					
No change	Monkey	Oral; 650 μg ml^−1^; 6–8 months	NAcc	α6β2*	109
		Oral; 650 μg ml^−1^; 8 months	VPu; DPu	α6β2*	110
		Oral; 650 μg ml^−1^; 3–6 months	NAcc	α6β2*	111
					
	Rat	Injection; 6.0 mg kg^−1^ per day; 2 weeks	Str; SC	β3*	105
			SC	α6*	
					
	Neuro-2a cell	Incubation; 50 μm; 24 h	—	α6β2*	102
					
Downregulation	Rat	Oral; 650 μg ml^−1^; 6 months	CPu; AcbC; AcbSh; SNPC; VTA	α6β2*	113
		Injection; 6.0 mg kg^−1^ per day; 2 weeks	Str	α6*	105
		Injection; 6.0 mg kg^−1^ per day; 2 weeks	Str; DLG; VLG	α6*	106
		Oral; 100 μg ml^−1^; 2 weeks	Str	α6β2*	107
		Oral; 25 μg ml^−1^; 2–3 months	NAcc	α6β2*	114
					
	Mouse	Oral; 300 μg ml^−1^; 1–6 weeks	Str	α6*	104
		Oral; 300 μg ml^−1^; 2 weeks	Str	α6β2*	107
		Injection; 0.125–4.0 mg kg^−1^ per h; 10 day	DLG; NAcc; Str; OT; VLG	α6β2*	108
					
	Monkey	Oral; 650 μg ml^−1^; 6 months	Str	α6*	112

Abbreviation: AcbC, core of nucleus accumbens; AcbSh, shell of nucleus accumbens; CPu, caudate putamen; DLG, dorsolateral geniculate; DPu, dorsal putamen; HEK, human embryonic kidney; NAcc, nucleus accumbens; Neuro, neuroblastoma; OT, olfactory tubercle; SC, superior colliculus; SN, substantia nigra; SNPC, pars compacta of substantia; Thal, thalamus; VLG, ventrolateral geniculate; VTA, ventral tegmental area mHb.
